# A200 HIGH-DEFINITION CHROMOENDOSCOPY RESULTS IN MORE SIGNIFICANT DYSPLASIA DETECTION THAN WHITE LIGHT ENDOSCOPY WITH RANDOM BIOPSIES IN ULCERATIVE COLITIS PATIENTS

**DOI:** 10.1093/jcag/gwac036.200

**Published:** 2023-03-07

**Authors:** T T Hoang, Y Leung, G Rosenfeld, B Bressler

**Affiliations:** Division of Gastroenterology, Department of Medicine, University of British Columbia, Vancouver, Canada

## Abstract

**Background:**

Ulcerative colitis (UC) is an inflammatory bowel disease that results in inflammation of the colonic mucosa, leading to abdominal pain, rectal bleeding, weight loss, and diarrhea. This chronic inflammation results in a 2.4-fold increased future risk of developing colorectal cancer (CRC) in UC patients compared to the general population. Thus, careful dysplasia screening modalities are required to prevent progression to CRC. Currently, both high-definition white light endoscopy with non-targeted biopsies (HD-WLR) and dye-spray chromoendoscopy (HDCE) are regularly used across Canada for dysplasia surveillance given existing research has been inconclusive regarding superiority of one particular method, and that recent guidelines do not suggest a preference.

**Purpose:**

The primary objective of this study was to determine which surveillance modality yielded a higher dysplasia detection rate in UC patients, both by calculating the total number of dysplastic lesions detected, as well as calculating the number of patients with at least one dysplastic lesion detected using either technique.

**Method:**

We conducted a single-centre retrospective chart review of 150 UC patients who underwent dysplasia surveillance at our site between January 2019-2021. We calculated the dysplasia detection rate of both techniques at the time of the first CRC screening colonoscopy.

**Result(s):**

Eighteen dysplastic lesions were detected in total, three by HD-WLR and fifteen by HDCE. Dysplasia was detected in 4% (3/75) and 14.5% (15/75) of UC patients by HD-WLR and HDCE respectively, with significantly fewer biopsies (4.44 + 4.3 vs 29.1 + 13.0) required. HD-WLR detected two polypoid and one non-polypoid lesion, while HDCE detected eleven polypoid and four non-polypoid lesions. No invisible dysplasia or colorectal cancer was detected. Screening was performed at 10.8 + 4.8 and 9.72 + 3.05 years following UC diagnosis for HDCE and HD-WLR respectively. Median withdrawal time was 9.0 + 2.7 min (HD-WLR) vs 9.6 + 3.9min (HDCE).

**Image:**

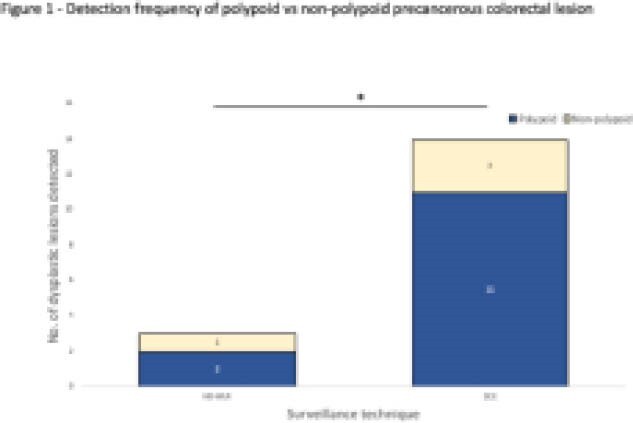

**Conclusion(s):**

HDCE resulted in higher dysplasia detection rates compared to HD-WLR in a UC patient population. Given the former technique is less tedious and costly, our findings suggest HDCE should be considered over HD-WLR for UC dysplasia surveillance.

**Please acknowledge all funding agencies by checking the applicable boxes below:**

None

**Disclosure of Interest:**

None Declared

